# What Drives Internet Entrepreneurial Intention to Use Technology Products? An Investigation of Technology Product Imagination Disposition, Social Support, and Motivation

**DOI:** 10.3389/fpsyg.2022.829256

**Published:** 2022-03-16

**Authors:** Tien-Chi Huang, Yi-Jin Wang, Hui-Min Lai

**Affiliations:** ^1^Department of Information Management, National Taichung University of Science and Technology, Taichung, Taiwan; ^2^Department of Business Administration, National Taichung University of Science and Technology, Taichung, Taiwan

**Keywords:** internet entrepreneurial intention, intrinsic and extrinsic motivation, social cognitive theory, imagination, social support, technology product

## Abstract

Technological products such as computer, communication, and consumer electronic products, apps, smart wearables, and streaming services have become inseparable from people’s lives. In technological fields of practice, imagination, creativity, innovation, and entrepreneurship may influence one another. A vivid imagination can generate creativity and trigger the entrepreneurial intention to “bring new things to the market.” This study aims to understand the formation of internet entrepreneurial intention to use technology products. Drawing on social cognitive theory, this study explores and empirically tests how technology product imagination disposition and social support impact internet entrepreneurial intention to use technology products. Drawing from self-determination theory, this study proposes and examines the mediating role of intrinsic (challenge and enjoyment) and extrinsic motivation (compensation and outward motivation) in the relationship between technology product imagination disposition and internet entrepreneurial intention, as well as the relationship between social support and internet entrepreneurial intention. We conducted a survey of 568 adults in Taiwan and used partial least squares to test our hypotheses. The results show the following: (1) Technology product imagination disposition is positively associated with challenge, enjoyment, compensation, and outward motivation. (2) Social support is positively associated with challenge, enjoyment, compensation, and outward motivation. (3) Challenge, enjoyment, and outward motivation are positively associated with internet entrepreneurial intention to use technology products. (4) Technology product imagination disposition intensifies internet entrepreneurial intention to use technology products by strengthening challenge, enjoyment, and outward motivation. Social support intensifies internet entrepreneurial intention by increasing challenge, enjoyment, and outward motivation. The partial mediation model represents a significant improvement in the total effect over the direct effect. We discuss the implications of these results for research and internet entrepreneurship practices.

## Introduction

To reduce the high unemployment rate in Taiwan, the government has formulated investment, employment, and entrepreneurship policies that have steadily drawn attention to entrepreneurship. Many colleges and universities in Taiwan have introduced entrepreneurship-related courses to strengthen awareness of innovation and entrepreneurship, to cultivate entrepreneurial ability, and to generally encourage entrepreneurship. Since the end of 2019, the COVID-19 pandemic has spread throughout the world, resulting in dramatic changes in industrial structures and deepening instability around the availability of jobs. The pandemic has stimulated the development of technology products, such as computers, communication devices, consumer electronic products, apps, wearable devices, films, platforms, and live broadcasts for online communication (Wang et al., 2020). For example, tracking devices were developed to ensure that people remain in self-quarantine in hotels, while virtual care platforms have been expanded to deliver remote care to patients and to reduce healthcare burdens during COVID-19 (Vuong et al., 2022).

According to a survey by Taipeiecon (Huang, 2021), which reports on entrepreneurship in Taiwan in 2020, entrepreneurs are mainly young and middle-aged men, and the mainstream trend is in science and technology entrepreneurship. The business opportunities opened up by the emerging technologies are pushing countries and enterprises around the world toward digital transformation. “internet entrepreneurship” is emerging as a practical approach (Lian and Yen, 2017), and it has advantages that the traditional entrepreneurial model does not. Digital technologies are able make entrepreneurial activities increasingly effective (Arvidsson and Mønsted, 2018), and they offer acceptable careers for the younger generation. The products that people design, develop, and distribute through technology are generally referred to as technology products. For example, Amazon, now an e-commerce giant, evolved from a regular online bookstore to become a top global power in the cloud market, e-commerce, and digital content market, creating countless technology products, such as Kindle and the Kindle Fire tablet. Another company, RD English, uses YouTube to distribute knowledge to its one million subscribers, finding a balance between entertainment and teaching while also developing technology products, such as themed videos and online English courses. Apple Watch is a technology product that combines mobile phone and app functions to enable people to monitor their health, anytime, anywhere, by measuring blood oxygen concentration, heart rate, sleep, exercise data, and by providing various safety and emergency functions, making life safer and more efficient. Wireless Bluetooth microphones are another technology product, which connect Bluetooth to a mobile device and transmit audio through a special Bluetooth protocol. These microphones are more convenient than wired microphones and can be used in many situations, such as in teaching and learning, games and entertainment, and in conference hosting. All these technology products are suitable for use in the internet entrepreneurial model, as they can be quickly disseminated and presented through the internet.

All this gives rise to an interesting question: What drives internet-based entrepreneurial intention to use technology products? Innovative and creative technology products usually result from a wealth of imagination. Imagination is the foundation of creativity (Vygotsky, 2004). People first imagine, and then they create. Technology products have become inseparable from people’s lives, and we can imagine that new technologies will continue to be developed in the next 10 or 20 years. However, very little research is being done to measure the role of imagination in the development of technology products. For this study, we revised the Technology Imagination Disposition Scale (Lin, 2019), which is a scale used to measure the individual’s tendency to imagine new technology products. Social cognitive theory (SCT) states that personal behavior will depend on both personal factors and environmental factors, and that these factors are related (Bandura, 1978). Environmental factors, such as the social and cultural environment, and personal factors, such as self-efficacy and creativity, will influence peoples’ entrepreneurial behavior (Nwosu et al., 2022). Entrepreneurship does not only involve personal behaviors but it is also a social activity. In addition to personality and skills, entrepreneurship will be impacted by factors such as family support and social relationships in the environment. We therefore added “social support” to the original scale as an environmental factor.

Many universities find that their new entrepreneurship-related courses are more ambitious than the entrepreneurial behaviors that derive from them. Thus, university instructors realize that there is still much to learn about how best to drive people’s internet entrepreneurial intention. Drawing from self-determination theory (SDT), two types of human motivation (intrinsic and extrinsic motivation) directly impact human behavior (Deci and Ryan, 1985). Motivation is an internal state that gives individuals direction and energy, and it makes them maintain behaviors or activities (Maehr and Meyer, 1997). Motivation should thus be considered an important construct in predicting entrepreneurship (Wang et al., 2016). This study adopts the scale of intrinsic and extrinsic motivation developed by Amabile et al. (1994). Intrinsic motivation has two secondary factors, challenge and enjoyment, and it includes self-determination, competence, work engagement, curiosity, and interest. Extrinsic motivation has two secondary factors, compensation and outward motivation, and it includes evaluation, recognition, competition, money, other external rewards, and the opinions of others. The following research questions were formulated:

RQ1. How do technology product imagination disposition, social support, intrinsic motivation (challenge and enjoyment), and extrinsic motivation (compensation and outward motivation) contribute to internet entrepreneurial intention to use technology products?
RQ2. How does technology product imagination disposition affect intrinsic motivation (challenge and enjoyment) and extrinsic motivation (compensation and outward motivation) and thereby contribute to increased internet entrepreneurial intention to use technology products?
RQ3. How does social support affect the intrinsic motivation (challenge and enjoyment) and extrinsic motivation (compensation and outward motivation) that contribute to increased internet entrepreneurial intention to use technology products?

## Theoretical Background and Hypotheses Development

### Internet Entrepreneurial Intention

The goal of entrepreneurship is to create a new business (Low and MacMillan, 1988; Amit and Zott, 2001). Thus, entrepreneurial intention is an important driver of entrepreneurial behavior (Zhao et al., 2005; Liñán and Chen, 2009). Internet entrepreneurship aims to create a new business through internet resources and technology, which is an extension of traditional entrepreneurship. Internet entrepreneurial intention is defined as the estimation of the possibility that a person will start and own a new e-commerce business (Wang et al., 2016; Chang et al., 2020). In this study, internet entrepreneurship more broadly includes business models developed through the internet, such as internet marketing, internet social media, website setup, live broadcasting, and app creation. Scholars have paid considerable attention to entrepreneurial intention, yet studies on internet entrepreneurial intention are still very limited. As shown in Table 1, few researchers have explored this issue from the perspective of personal traits, personal motivation, and surrounding environmental factors simultaneously—which are closely related to an individual’s internet entrepreneurial intention.

**TABLE 1:** Summary of previous research on internet entrepreneurial intention.

Study	Samples	Determinants of internet entrepreneurial intention
Chang et al., 2020	760 college students	Cyber-entrepreneurial self-efficacy (+), positive thinking (ns)
Chang et al., 2018	146 undergraduate students with entrepreneurial role models and 133 without	*For the with entrepreneurial role models group:* Cyber-entrepreneurial self-efficacy (+), goal commitment (ns); *For the without entrepreneurial role models group:* Cyber-entrepreneurial self-efficacy (ns), goal commitment (+)
Wang et al., 2016	450 final-year undergraduate students in Taiwan	Intrinsic cyber-entrepreneurial motivation (+), extrinsic cyber-entrepreneurial motivation (+)
Ismail et al., 2012	155 university students	Self-employment (−), risk attitude (ns), need for achievement (ns), internal control (ns)
Millman et al., 2010	303 undergraduate and graduate students	Gender (−): Male students exhibit more internet entrepreneurial intention than female students

*(+) positive effect; (−) negative effect; (ns) non-significant.*

### Technology Product Imagination Disposition

The personal trait examined in this study is technology product imagination disposition, a concept from SCT. Imagination is an innate human ability that is also the basis of creative activities (Ho et al., 2013). Imagination enables people to surpass experience and create new possibilities. It is the most important aspect of cognitive ability for learning (Heath, 2008). “Technology” is the use of knowledge, resources, and skills to solve life problems or to expand self-capacity as a tool for adapting to the environment (Mohr et al., 2010). Through the process of imagination, students can develop novel ideas and then produce products or specific objects (Eckhoff and Urbach, 2008). There is very little literature on technological imagination, most of which is related to scientific imagination. The present study focused on exploring technology products in the context of internet entrepreneurial intentions. Willingness to use technology products for internet entrepreneurship differs from Lin’s (2019) definition. “Technology product imagination disposition” refers to the habit of thinking about technology products used in the future and the ability to combine old experiences and existing objects to imagine and create technologies that do not exist and generate positive emotions.

Technology product imagination disposition is a personal trait. Therefore, we infer that this trait is positively related to personal challenges and enjoyment motivations. Individuals with a higher technology product imagination disposition may enhance their extrinsic motivation for internet entrepreneurship; that is, they want to obtain material or financial rewards or be recognized and affirmed by others. Therefore, we propose the following hypothesis:

H1: Technology product imagination disposition is positively associated with (a) challenge motivation, (b) enjoyment motivation, (c) compensation motivation, and (d) outward motivation.

### Social Support

The environmental factor examined in this study was social support, a concept from SCT. Social support was first proposed by Caplan (1974). It is believed that social support refers to individuals meeting basic social needs, emotions, personal identity, and values through interactions between others or groups, and social support is divided into substantive and non-substantive support. Social support comes from a wide range of sources. The support of a spouse, family, classmates, friends, teachers, or mentors can reduce pressure on some events and enable individuals to deal flexibly with events and maintain a good state of physical and mental adjustment (Heller and Swindle, 1983). Social support can be roughly divided into three dimensions: tangible support, emotional support, and informational support. Tangible support is the most direct resource, which refers to direct assistance, such as practical action and material, to help supportees understand and solve problems. Emotional support is love, care, empathy, listening, comfort, encouragement, or trust. Informational support includes giving suggestions or advice to help supportees understand and solve problems. In this study, Krause and Markides (1990) social support scale is used as the basis; tangible support, emotional support, and informational support are taken as the main dimensions of social support, and the study extends Cobb’s (1976), Gottlieb’s (1978), and Barrera et al.’s (1981) work. We define social support as feeling and receiving substantive, emotional, and information care and assistance from others when individuals need help. According to Krause and Markides (1990), social support is a second-order reflective construct. In the present study, social support included three first-order latent variables: informational support, tangible support, and emotional support.

When entrepreneurs have good social support, they experience a better entrepreneurial environment and better interpersonal relationships and are ready for entrepreneurship. Therefore, it is possible to strengthen an individual’s intrinsic motivation, that is, the individual’s belief that he or she can challenge himself or herself and obtain satisfaction in the process of internet entrepreneurship. Social support may also strengthen an individual’s extrinsic motivation—that is, an individual’s belief that he or she can get a monetary reward or be recognized by others in the process of internet entrepreneurship. Thus, we propose the following hypothesis:

H2: Social support is positively associated with (a) challenge motivation, (b) enjoyment motivation, (c) compensation motivation, and (d) outward motivation.

### Intrinsic and Extrinsic Motivation

The personal motivations examined in this study are intrinsic and extrinsic motivation, concepts from SDT. Personal behavior is determined by intrinsic and extrinsic motivation (Teo et al., 1999). Wang et al. (2016), in the context of e-commerce, defined intrinsic motivation for internet entrepreneurship as the creation of a new e-commerce business in the process of implementation to obtain inherent incentives. Extrinsic motivation for internet entrepreneurship was defined as the creation of a new e-commerce business to obtain external rewards. Based on Amabile et al.’s (1994) study, we divide internet entrepreneurial motivation into intrinsic motivation and extrinsic motivation for internet entrepreneurship and subdivide internet entrepreneurial motivation into secondary factors. Based on Amabile et al. (1994) and Wang et al. (2016), we defined intrinsic motivation for internet entrepreneurship as individuals’ use of technology products for internet entrepreneurship to obtain internal rewards. We defined extrinsic motivation for internet entrepreneurship as individuals’ use of technology products for internet entrepreneurship behavior to obtain external rewards.

Shane et al. (2003) believed that the development of entrepreneurial theory needed to consider people’s motivations for making entrepreneurial decisions, and that differences in motivation also affect the entrepreneurial process. When people are willing to accept challenges and think that internet entrepreneurship can obtain enjoyment and satisfaction or get external rewards and recognition, they are more likely to have high internet entrepreneurial intention to use technology products. Therefore, we propose the following hypothesis:

H3: (a) Challenge motivation, (b) enjoyment motivation, (c) compensation motivation, and (d) outward motivation are positively associated with people’s internet entrepreneurial intention to use technology products.

### Mediating Role of Intrinsic and Extrinsic Motivation

Seelig (2015) defined the concepts of imagination, creativity, innovation, and entrepreneurship, showed how they affect each other, and unveiled that imagination leads to creativity, creativity leads to innovation, and innovation leads to entrepreneurship. Therefore, technology product imagination disposition is positively associated with people’s internet entrepreneurial intention to use technology products. Wang et al. (2016) pointed out that personality traits strengthen intrinsic and extrinsic motivations and then affect internet entrepreneurial intention. To summarize, motivation may mediate the effect of personality and internet entrepreneurial intention. Thus, we expect that the impact of technology product imagination disposition on internet entrepreneurial intention to use technology products not only can be measured directly but also can be quantified by examining several intervening intrinsic (e.g., challenge and enjoyment) and extrinsic (e.g., compensation and outward) motivations. Therefore, we posit the following hypotheses:

H4a: The relationship between technology product imagination disposition and people’s internet entrepreneurial intention to use technology products is partially mediated by challenge motivation.
H4b: The relationship between technology product imagination disposition and people’s internet entrepreneurial intention to use technology products is partially mediated by enjoyment motivation.
H4c: The relationship between technology product imagination disposition and people’s internet entrepreneurial intention to use technology products is partially mediated by compensation motivation.
H4d: The relationship between technology product imagination disposition and people’s internet entrepreneurial intention to use technology products is partially mediated by outward motivation.

Sahban et al. (2016) pointed out that social support positively affects entrepreneurial intentions. Therefore, the importance of social support for entrepreneurship, the degree of social support for entrepreneurship, and the degree to which opportunities and resources are provided may affect people’s internet entrepreneurial intention to use technology products. Thus, we hypothesize that social support is positively related to internet entrepreneurial intention to use technology products. Furthermore, Deci and Ryan’s (1985) SDT suggests that people are likely to be willing to engage in behaviors for intrinsic versus extrinsic motivation. When people have intrinsic and extrinsic motivations, in addition to social support, they have internet entrepreneurial intention. In other words, internet entrepreneurial intention to use technology products is based on their intrinsic and extrinsic motivations, which, in turn, benefit from social support. Therefore, we propose the following hypotheses:

H5a: The relationship between social support and people’s internet entrepreneurial intention to use technology products is partially mediated by challenge motivation.
H5b: The relationship between social support and people’s internet entrepreneurial intention to use technology products is partially mediated by enjoyment motivation.
H5c: The relationship between social support and people’s internet entrepreneurial intention to use technology products is partially mediated by compensation motivation.
H5d: The relationship between social support and people’s internet entrepreneurial intention to use technology products is partially mediated by outward motivation.

**FIGURE 1:**
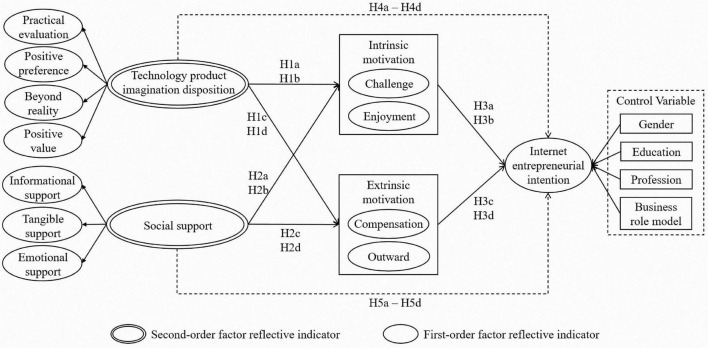
Research model.

The research model and hypotheses are illustrated in Figure 1.

## Materials and Methods

### Procedure

This study had three main phases. First, the questionnaire was based on well-established scales from previous behavioral and entrepreneurial research and was then pre-tested by four experts in the fields of information management, e-learning, internet behavior, and entrepreneurship. The semantics of the questionnaire were corrected, and inappropriate items were deleted. Second, a pilot test was conducted from June 1, 2020, to June 30, 2020. A total of 135 valid questionnaires were obtained. A second-order model of technology product imagination disposition was constructed and demonstrated (Wang et al., 2020). Third, a formal survey was conducted from August 10, 2020, to March 31, 2021. An online questionnaire surveyed adults over the age of 18.

### Measurement

Most of the questionnaire items were based on previous research. The definitions of the variables are shown in Table 2. A five-point Likert scale was used for responses: 1 (strongly disagree), 2 (disagree), 3 (neutral), 4 (agree), and 5 (strongly agree). We also included four control variables that might affect internet entrepreneurial intention to use technology products: gender, education, occupation, and business role models. First, there are differences in entrepreneurial intentions between men and women (Sahban et al., 2016). Therefore, we inferred that gender affects internet entrepreneurial intention. Gender was dummy coded as 0 (male) and 1 (female). Second, Quan (2012) pointed out that education might have an impact on an individual’s willingness to discover new opportunities; thus, education might contribute to impulsive internet entrepreneurial intention. Education level was coded as 1 (less than junior high school), 2 (senior high school), 3 (junior college), 4 (university), or 5 (more than graduate school). Third, internet entrepreneurial intention might be affected by student and non-student status; thus, occupation was a dummy variable coded as 0 (student) and 1 (non-student). Finally, business role models refer to family, teachers, and friends. The results show that the existence of business role models has an impact on students’ internet entrepreneurial self-efficacy and internet entrepreneurial intention (Chang et al., 2018). Business role models was dummy coded as 0 (Yes) and 1 (No).

**TABLE 2:** Definitions of variables.

Variable	Definition	References
Technology product imagination disposition	Individuals have a habit of thinking about technology products to be used in the future and can imagine them in combination with experience and existing items, create technology products that do not exist today, and produce positive emotions	Lin, 2019
Informational support	Supporters provide information such as suggestions or advice to help supportees understand and solve problems	Krause and Markides, 1990
Tangible support	Supporters provide direct assistance in practical actions and material and financial matters to help supportees understand and solve problems	
Emotional support	Supporters provide emotional support such as love, care, empathy, and trust to help supportees face and solve problems	
Challenge motivation	The extent to which individuals prefer to seek out challenging, complex, and difficult tasks for internet entrepreneurship	Amabile et al., 1994
Enjoyment motivation	The extent to which individuals prefer to have enjoyment, interest, and satisfaction for internet entrepreneurship	
Compensation motivation	The extent to which individuals are concerned about material or monetary rewards for internet entrepreneurship	Amabile et al., 1994
Outward motivation	The extent to which individuals are concerned about recognition and affirmation by others	
Internet entrepreneurial intention to use technology products	It is estimated that a person will start a new internet business using technology products	Liñán and Chen, 2009

### Revision of the Technology Product Imagination Disposition Scale

The Technology Product Imagination Disposition Scale was developed based on Lin’s (2019) Technology Imagination Disposition Scale and is divided into six dimensions. We defined technology products as software and hardware technology (Wang et al., 2020), including apps, websites, self-media, videos, etc., as well as various technologies encountered in daily life (such as wearable devices and wireless Bluetooth microphones). In this study, questionnaire items that were not easy to understand were deleted after the pre-test. A total of four questionnaire items were deleted. Several remaining questions were adjusted and revised, and a total of 20 questionnaire items were retained. Next, we used SPSS 23.0 to perform exploratory factor analysis and reanalyzed the measurement dimensions. The results are shown in Supplementary Appendix A. The Kaiser–Meyer–Olkin (KMO) value was 0.89 (>0.5), and the Bartlett test was 9942.956 (*p* < 0.05), considering that the eigenvalues were greater than 1 (Horn, 1965), resulting in four factors. We deleted three items because the factor loadings were below 0.5. Seventeen items remained, and the cumulative total explained variance was 62.19%. The four factors were (1) practical evaluation (the benefits that individuals can often imagine the possibility, practicality, mass production, acceptance, and widespread use of technology products); (2) positive preference (an individual’s positive emotions about and preferences for imaginary technology products, which will produce happy and interesting feelings and try to improve their own imagination); (3) beyond reality (improving imagination for individuals to break through limitations in the real world and construct unprecedented lifestyles or technology products as well as imagine plots or human characteristics that transcend the real characteristics from their ideas); and (4) positive value (an individual’s evaluation of the value of imaginary technology products, and the degree to which they believe that the imaginary technology products are beneficial for work and life). Wang et al. (2020) named positive value “attitude.”

The item analysis included a comparison of extreme groups, homogeneity testing, and internal consistency. Extreme groups were compared to evaluate the difference between 27% and 73% of the total score of the sampling scale. The difference in the results is called the critical ratio (CR). An independent sample *t*-test was used to delete items whose CR was not greater than 3.29 (*p* < 0.001) and did not reach a level of significance (*p* > 0.05; (Wolman, 1989). The homogeneity test uses Pearson item-scale correlations. Item internal consistency is satisfactory if the item-scale correlation coefficients are higher than 0.4 (Ware and Gandek, 1998) and reach statistical significance (*p* < 0.05). If not, the items are deleted. The item analysis results for technology product imagination disposition are shown in Supplementary Appendix B. The CR values of all items reached significance. The practical evaluation ranged from 7.384 to 10.179, the positive preference ranged from 6.935 to 8.903, the beyond reality ranged from 5.925 to 8.977, and the positive value ranged from 3.788 to 6.968. The corrected item–total correlation of the 18^th^ item was lower than the cut-off value of 0.4; however, using at least three items for measuring one variable is better. Thus, the 18^th^ item was retained. Finally, Cronbach’s alpha of the total scale was examined to evaluate the reliability of the scale. Cronbach’s alpha of the total scale was 0.903, which was higher than the recommended value of 0.7 (Nunnally, 1978), and Cronbach’s alpha after the deletion of each item did not exceed the total scale. Thus, all 17 items were retained.

Then, we used AMOS 23.0 to perform confirmatory factor analysis (CFA) of the model to examine the model fit of the Technology Product Imagination Disposition Scale. As shown in Table 3, most of the fit indices in the first-order single-factor model and the first-order four-factor orthogonal model did not reach the ideal value. Overall, the first-order four-factor oblique model and the second-order single-factor model could be adapted to the sample data, and the fit indices were good. From the point of view of model simplification, the Akaike information criterion (AIC) was applicable to the selection of a competitive model (Hu and Bentler, 1995). According to Hu and Bentler’s (1999) cut-off criteria for fit indexes, the second-order single-factor model had a good fit: χ^2^ = 171.831, *p* < 0.001. The standardized root mean squared residual (SRMR) was 0.061, the root mean square error of approximation (RMSEA) was 0.061, the Tucker–Lewis index (TLI) was 0.921, the comparative fit index (CFI) was 0.934, the factor loadings were between 0.54 and 0.77, and the AIC was relatively small, which can achieve the principle of model simplicity. Therefore, the second-order single-factor model was used as the CFA verification model and the technology product imagination disposition model as a second-order reflective construct.

**TABLE 3:** The fit indices of competing models of the Technology Product Imagination Disposition Scale.

Model fit indices	Criteria	Null model	First-order single-factor model	First-order four-factor orthogonal model	First-order four-factor oblique model	Second-order single-factor model
Degrees of freedom (*df*)		136	119	119	114	115
**Absolute fit indices**						
χ^2^ value (*p*)		991.028 (0.000)	268.709 (0.000)	354.553 (0.000)	230.292 (0.000)	171.831 (0.000)
RMR	<0.05	0.254	0.055	0.202	0.399	0.046
RMSEA	<0.08	0.217	0.097	0.122	0.087	0.061
SRMR	<0.08	0.3498	0.759	0.2798	0.2118	0.0611
GFI	>0.9	0.312	0.800	0.751	0.849	0.875
AGFI	>0.9	0.226	0.743	0.680	0.798	0.833
**Value-added fit indices**						
NFI	>0.9		0.729	0.642	0.768	0.827
IFI	>0.9		0.828	0.730	0.867	0.935
TLI	>0.9		0.800	0.685	0.838	0.921
CFI	>0.9		0.825	0.725	0.864	0.934
**Simple fit indices**						
PGFI	>0.5	0.278	0.622	0.584	0.633	0.658
PNFI	>0.5		0.638	0.562	0.643	0.699
PCFI	>0.5		0.722	0.634	0.724	0.789
χ^2^/*df*	<3	7.287	2.258	2.979	2.020	1.494
AIC	The smaller, the simpler	1025.028	336.709	422.553	308.292	247.831

*RMR, root mean square residual; RMSEA, root mean square error of approximation; SRMR, standardized root mean squared residual; GFI, goodness-of-fit index; AGFI, adjusted goodness-of-fit index; NFI, normed fit index; IFI, incremental fit index; TLI, Tucker–Lewis index; CFI, comparative fit index; PGFI, parsimonious goodness-of-fit index; PNFI, parsimonious normed fit index; PCFI, parsimonious comparative fit index; AIC, Akaike information criterion.*

### Participants

The main survey participants were adults older than 18 years in Taiwan, and the questionnaire was distributed and collected online. We selected adults older than 18 years old as participants because the sample is similar to that used in the well-known Global Entrepreneurship Observation Report. People were invited via social media to fill out the questionnaire online. A total of 589 questionnaires were collected, and 21 participants younger than 18 years were deleted. In total, 568 valid questionnaires were completed by students and non-students. Among the participants, students accounted for about 68.1%, and non-students accounted for about 31.9%; men accounted for about 46%, and women accounted for about 54%. Detailed demographic information is shown in Table 4.

**TABLE 4:** Demographic information of respondents (*N* = 568).

Demographic variable	Count (%)	Demographic variable	Count (%)
Occupation		Age	
Student	387 (68.1%)	18–25 years	455 (80.1%)
Non-student	181 (31.9%)	26–30 years	57 (10.0%)
Gender		31–35 years old	23 (4.00%)
Male	261 (46.0%)	36–40 years old	17 (3.00%)
Female	307 (54.0%)	41 years or older	16 (2.80%)
Education level		Business role models	
Senior high school	5 (0.9%)	Yes	396 (69.7%)
Junior college	5 (0.9%)	No	172 (30.3%)
University	323 (56.9%)		
More than graduate school	235 (41.4%)		

### Data Analysis

Data analysis was conducted using SmartPLS 3.0 (Ringle et al., 2015) to test the proposed models in two steps: The reliability and validity of the items were examined to validate the measurement model, and partial least squares (PLS) was used to examine the structural model for the significance and explanatory power of the hypotheses. PLS is appropriate because it allows non-normality and small to medium sample sizes (Chin et al., 2003) and aims to maximize the proportion of variance in the dependent variable explained by all predictor variables (Easley et al., 2003).

## Results

### Reliability and Validity

In terms of reliability, the Cronbach’s alpha of each variable was between 0.797 and 0.937, reaching the threshold of 0.7 (Nunnally, 1978). The CR was between 0.845 and 0.950, which also exceeded the threshold (Fornell and Larcker, 1981), indicating that the scale had good reliability, as shown in Supplementary Appendix C. In terms of convergent validity, the factor loadings of all items should be greater than the 0.5 cut-off value (Hair et al., 2006), and the average variance extracted (AVE) should be greater than 0.5 (Fornell and Larcker, 1981). As shown in Supplementary Appendix C, the AVEs of all variables reached the 0.5 cut-off value, except for technology product imagination disposition, social support, and outward motivation. The CR of all variables was greater than 0.6. According to Fornell and Larcker (1981), when the AVE is less than 0.5, if the CR of the variables is greater than 0.6, then convergent validity is still demonstrated. The results show acceptable convergent validity. In terms of discriminant validity, the square root of the AVEs should be greater than the correlation coefficient between the variables. As shown in Table 5, the results demonstrate good discriminant validity.

**TABLE 5:** Discriminant validity.

Variables	1	2	3	4	5	6	7
1. Internet entrepreneurial intention to use technology products	**0.890**						
2. Social support	0.072	**0.699**					
3. Technology product imagination disposition	0.433	0.333	**0.687**				
4. Challenge motivation	0.504	0.276	0.492	**0.752**			
5. Enjoyment motivation	0.501	0.312	0.505	0.747	**0.758**		
6. Compensation motivation	0.319	0.350	0.420	0.439	0.485	**0.755**	
7. Outward motivation	0.359	0.385	0.410	0.417	0.496	0.623	**0.665**

*The bold numbers in the diagonal row are square roots of the average variance extracted.*

### Hypotheses Testing

A bootstrapping procedure with 5,000 resamples in SmartPLS 3.0 was used to assess the significance level of the hypothesized paths. Technology product imagination disposition was positively associated with challenge motivation (β = 0.450, *p* < 0.001), enjoyment motivation (β = 0.451, *p* < 0.001), compensation motivation (β = 0.342, *p* < 0.001), and outward motivation (β = 0.318, *p* < 0.001); thus, H1a, H1b, H1c, and H1d were supported. Social support was positively associated with challenge motivation (β = 0.126, *p* < 0.01), enjoyment motivation (β = 0.162, *p* < 0.001), compensation motivation (β = 0.236, *p* < 0.001), and outward motivation (β = 0.279, *p* < 0.001); H2a, H2b, H2c, and H2d were supported. H3a was supported, as challenge motivation was positively associated with people’s internet entrepreneurial intention to use technology products (β = 0.249, *p* < 0.001). H3b was also supported, as the association between enjoyment motivation and internet entrepreneurial intention to use technology products was positive and statistically significant (β = 0.166, *p* < 0.05). H3c, which stated that compensation motivation is positively associated with people’s internet entrepreneurial intention to use technology products, was not supported (β = 0.019, *p* > 0.05). Outward motivation was positively associated with people’s internet entrepreneurial intention to use technology products (β = 0.139, *p* < 0.001); thus, H3d was supported.

Furthermore, when challenge, enjoyment, compensation, and outward motivation acted as mediators in the relationship between technology product imagination disposition and internet entrepreneurial intention, a significant indirect effect on challenge (β = 0.112, *t* = 3.972, *p* < 0.001), enjoyment (β = 0.075, *t* = 2.640, *p* < 0.01), and outward motivation (β = 0.044, *t* = 2.490, *p* < 0.05) was found. The direct effects were also positive and statistically significant for technology product imagination disposition on internet entrepreneurial intention (β = 0.211, *p* < 0.001); thus, partial mediation was present. Therefore, H4a, H4b, and H4d were supported. However, no mediation was found for compensation, as the direct effect was positive and statistically significant, but the indirect effect was not statistically significant (β = 0.006, *t* = 0.018, *p* > 0.05). Thus, H4c was not supported.

When challenge, enjoyment, compensation, and outward motivation acted as mediators in the relationship between social support and internet entrepreneurial intention, a statistically significant indirect effect on challenge (β = 0.031, *t* = 2.175, *p* < 0.05), enjoyment (β = 0.027, *t* = 2.030, *p* < 0.05), and outward motivation (β = 0.039, *t* = 2.205, *p* < 0.05) was found. Surprisingly, the direct effect of social support on internet entrepreneurial intention was negative and statistically significant (β = –0.190, *p* < 0.001); thus, H5a, H5b, and H5d were not supported. No mediation was found for compensation, as the direct effect was negative and statistically significant, and the indirect effect was not statistically significant (β = 0.004, *t* = 0.354, *p* > 0.05). Therefore, H5c was not supported. The mediation effect results are shown in Table 6.

**TABLE 6:** Test of mediation effects.

	Independent variable	Mediator	Dependent variable	Direct effect	Indirect effect	Total effect	Mediation	Outcome
H4a	TPID	CH	IEI	0.211***	0.112***	0.234***	Partial	Supported
H4b	TPID	E	IEI	0.211***	0.075**	0.234***	Partial	Supported
H4c	TPID	C	IEI	0.211***	0.006	0.234***	None	Not supported
H4d	TPID	O	IEI	0.211***	0.044*	0.234***	Partial	Supported
H5a	SS	CH	IEI	–0.190***	0.031*	0.100***	Partial	Not supported
H5b	SS	E	IEI	–0.190***	0.027*	0.100***	Partial	Not supported
H5c	SS	C	IEI	–0.190***	0.004	0.100***	None	Not supported
H5d	SS	O	IEI	–0.190***	0.039*	0.100***	Partial	Not supported

*p < 0.05, **p < 0.01, ***p < 0.001.

*TPID, Technology Product Imagination Disposition; SS, social support; CH, challenge motivation; E, enjoyment motivation; C, compensation motivation; O, outward motivation; IEI, internet entrepreneurial intention to use technology products.*

**FIGURE 2:**
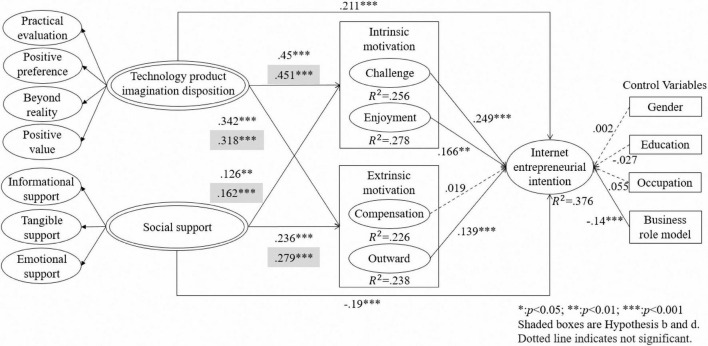
Results of partial least squares analysis.

The control variables—gender, education, occupation, and business role models—were included in the statistical analysis of the model. Gender (β = 0.002, *p* = 0.945), education (β = –0.027, *p* = 0.416), and occupation (β = 0.055, *p* = 0.126) were not associated with people’s internet entrepreneurial intention to use technology products, while business role models (β = –0.140, *p* < 0.001) was. The explanatory power of the structural model was evaluated from the *R*^2^ value in the variables: 25.6% variance in challenge, 27.8% variance in enjoyment, 22.6% variance in compensation, 23.8% variance in outward motivation, and 37.6% variance in internet entrepreneurial intention. The *R*^2^ values were above the 10% criterion (Falk and Miller, 1992), indicating that the proposed model was satisfactory in explaining the variance in challenge motivation, enjoyment motivation, compensation motivation, outward motivation, and internet entrepreneurial intention to use technology products. Figure 2 displays the path coefficients and their significance in the structural model.

## Discussion and Implications

### Discussion of Findings

In this study, we examined the relationships among technology product imagination disposition, social support, intrinsic motivation, extrinsic motivation, and internet entrepreneurial intention to use technology products. The major findings are as follows: First, some people seem to be driven by key personality traits, such as imagination, in their entrepreneurial intentions (Çelik et al., 2020). The data indicated that technology product imagination disposition is positively associated with internet entrepreneurial intention to use technology products. The findings of this study are consistent with Kier and McMullen (2018) research results that people with imagination have a higher number of entrepreneurial ideas. Additionally, the effects of technology product imagination disposition can be indirect. We showed that challenge, enjoyment, and outward motivation are mediators between technology product imagination disposition and internet entrepreneurial intention to use technology products. Specifically, technology product imagination disposition intensifies internet entrepreneurial intention by increasing challenge, enjoyment, and outward motivation. These results not only confirm the need for intrinsic and extrinsic motivations in internet entrepreneurship but also elaborate their mediating roles.

Second, compensation motivation is not positively related to internet entrepreneurial intention to use technology products. A possible explanation is that the entrepreneurial environment in Taiwan makes individuals feel that the attraction of external rewards is low. The existing literature (Vuong, 2018) has discussed the dilemma between cost consideration and value appreciation by scientists. It may take a long time for society to realize the true value of entrepreneurs’ technology product ideas. In such contexts, capital incentives and loan conditions provided by government agencies may not be sufficient, or the intention of internet entrepreneurs is not to get more rewards but to achieve their own life goals.

Third, the results also reveal that technology product imagination disposition and social support are key antecedents of intrinsic and extrinsic motivations. When individuals have a higher tendency to imagine technology products, they are more willing to flexibly apply and imagine the products they have created and have more complete and comprehensive conceptions and ideas, which will strengthen the challenge and enjoyment of intrinsic motivation and the outward motivation of extrinsic motivation. The results support our hypotheses and provide additional insights into how technology product imagination disposition and social support may be utilized to strengthen the motivation to increase internet entrepreneurial intention.

Finally, the results show that social support is negatively associated with internet entrepreneurial intention to use technology products, which is inconsistent with previous studies on the impact of social support on entrepreneurial intention (Sahban et al., 2016; Molino et al., 2018). One possible explanation for the negative relation between social support and internet entrepreneurial intention to use technology products may be that social support used in previous research referred to the support of family and friends; in particular, the definition of social support in the study conducted by Molino et al. (2018) focused on emotional support, excluding the material level. In the present study, social support included bosses, colleagues, relatives, friends, family, and peer friends. These supports include emotional, tangible, and informational support. Another possible explanation may be that the participants were from Taiwan and thus differed from those in previous research conducted in Italy and Indonesia, resulting in cultural differences. According to the Global Entrepreneurship Observation 2020/2021 Reports (GEM, 2021), among the indicators for assessing personal entrepreneurship, Taiwanese adults lag behind other countries, indicating that Taiwanese adults are afraid of the risks and uncertainties of entrepreneurship and are concerned that they feel that entrepreneurship opportunities and entrepreneurial capabilities are not highly evaluated, showing weak self-confidence. When they have more support and higher expectations from Taiwanese environmental supporters, but they cannot obtain a substantial level of financial support, they may feel pressure and lack self-confidence in their intentions. As a result, social support leads to low internet entrepreneurial intention. Additionally, the effects of social support can be indirect. We showed that challenge, enjoyment, and outward motivation act as mediators between social support and internet entrepreneurial intention to use technology products. Specifically, social support intensifies internet entrepreneurial intention by increasing challenge, enjoyment, and outward motivation. Thus, the partial mediation model represents a statistically significant improvement in the total effect (β = 0.100, *p* < 0.001) over the direct effect (β = –0.190, *p* < 0.001) and further supports the importance of challenge, enjoyment, and outward motivation in the formation of internet entrepreneurial intention to use technology products.

### Theoretical Implications

This study makes four contributions to theory. First, this study focused on technology products. In this era of rapid technological development, technology products are indispensable to humankind. Imagination has always been the foundation of creativity. We developed and empirically validated a multidimensional scale to assess people’s technology product imagination disposition for four components (i.e., practical evaluation, positive preference, beyond reality, and positive value) and linked them together through a second-order factor structure. This scale offers a more comprehensive measure of technology product imagination disposition, and it can be used as a reference for understanding the research topics of internet entrepreneurial intention to use technology products in the future.

Second, regarding the entrepreneurship literature, most previous studies focused on entrepreneurial intentions. Studies on internet entrepreneurial intentions are still lacking. The development of the internet has contributed to the internet business model becoming the model of choice for entrepreneurs. Therefore, understanding the formation of internet entrepreneurial intention is important. Behavior is generated depending on personal factors and environmental factors. This study contributes to the theoretical understanding of the internet entrepreneurial intention to use technology products. In particular, drawing on SCT and SDT, a comprehensive model including technology product imagination disposition, intrinsic motivation, extrinsic motivation, and social support was developed and examined.

Third, this study has not only explored the nature of four types of motivation—challenge, enjoyment, compensation, and outward motivation affecting internet entrepreneurial intention to use technology products—but also found that challenge, enjoyment, and outward motivation mediate the relationship between technology product imagination disposition and internet entrepreneurial intention to use technology products as well as the relationship between social support and internet entrepreneurial intention to use technology products. These findings add insights to previous internet entrepreneurship literature, which often emphasizes the effect of intrinsic and extrinsic motivation on internet entrepreneurial intention (Wang et al., 2016), but not their mediating role.

Finally, among the environmental factors in this study, social support had a significant negative effect on the internet entrepreneurial intention to use technology products, but it indirectly affected internet entrepreneurial intention to use technology products through challenge, enjoyment, and outward motivation. The study clarified the role of intrinsic and extrinsic motivations and suggests that challenge, enjoyment, and outward motivation are important mediating variables, which can play a critical role in the model of internet entrepreneurial intention to use technology products. These findings complement the internet entrepreneurship literature, which revealed that social support intensifies internet entrepreneurial intention to use technology products by increasing challenge, enjoyment, and outward motivation.

### Practical Implications

This study makes four contributions to practice. First, the results show that technology product imagination disposition is positively associated with internet entrepreneurship intention to use technology products. Kier and McMullen (2018) propose that imagination is the essential requirement in entrepreneurship and for generating entrepreneurial ideas. However, entrepreneurship may be inhibited by a lack of funds, resources, knowledge, skills, or by legal issues (Nwosu et al., 2022). We suggest that schools need to strengthen their training for students in professional subjects and legal issues, and that institutions outside of schools should set up courses relating to the development of imagination and creativity, as well as vocational training courses for job seekers. In these ways, learning opportunities would be provided through a number of courses to enable the public to understand entrepreneurship and the relevant skills. Moreover, greater attention should be paid to benefitting the environment, as current business practice needs to build corporate responsibility regarding the importance of protecting the environment. Institutions and universities should include this issue in their imagination and creativity courses.

Second, social support is shown to be negatively associated with internet entrepreneurial intention to use technology products. Therefore, it can be inferred that social support leads Taiwanese adults to feel less positive about their internet entrepreneurial intentions, and they may even experience social pressure that reduces their internet entrepreneurial intention to use technology products. As Vuong et al. (2022) suggest, cooperation among involved parties, for example, those with expertise from outside of the discipline and those from within the discipline, can be effective in supporting the process of creativity by helping people assess the originality and effectiveness of their designs. We recommend that schools and corporate organizations work together in assisting students who are taking entrepreneurship courses and thereby, accelerate the development of effective entrepreneurial ideas. Additionally, social support can increase internet entrepreneurial intention to use technology products by strengthening the challenge and enjoyment motivation. We suggest that individuals should be guided to establish interests that enhance their self-confidence. For example, schools can promote enterprise internships and community activities that increase students’ ability to respond to challenges with self-confidence and thereby, stimulate their internet entrepreneurial intention to use technology products after graduation.

Third, the results reveal a mediation effect by outward motivation between social support and internet entrepreneurial intention to use technology products, which shows the importance of seeking recognition and obtaining others’ approval. To enhance outward motivation, schools could create professional training courses. Alternatively, government agencies could hold entrepreneurship competitions that provide forums to guide individuals in developing their own entrepreneurial interests and through which they can receive social recognition. In this way, their internet entrepreneurial intention to use technology products could be increased.

Finally, this study found that business role models increase the individual’s internet entrepreneurial intention to use technology products. Role models can establish the means for an open dialog through which to share ideas and findings and to facilitate the absorbing, filtering, evaluating, connecting, and comparing of information in order to generate effective and useful insights (Vuong et al., 2022). Schools and government agencies could hold relevant competitions and lectures relating to internet entrepreneurship to increase people’s direct contact with entrepreneurs, especially entrepreneurs who are close to the people’s lives, such as older people who started a business after graduation, or to hear the experience of famous domestic entrepreneurs. This would help to improve internet entrepreneurial intention to use technology products.

### Limitations and Future Research Directions

The present study has several limitations. First, the data were collected from adults age 18 years and older in Taiwan; thus, the results may not be generalizable to other countries. It would be interesting to use the model in other countries to test whether it can be generalized in a broader context. Second, in this study, technology products were defined as 3C products, apps, smart wearables, videos, platforms, and live broadcasts. The scope of the definitions may be too broad. In the future, the scope can be narrowed to discuss specific types of technology products, such as Bluetooth products, apps, or live broadcasting. Third, this study did not distinguish between student and non-student participants. The number of student participants was higher than that of non-students. Students and non-students have different social experiences and may have different thoughts and ideas. However, this limitation might be controlled by including occupation as a control variable. Future researchers could add a sample size and use group analysis for comparison. Fourth, the research model included technology product imagination disposition, personal motivation, and social support for understanding the formation of internet entrepreneurial intention to use technology products. To extend the research model, additional perspectives such as Vuong and Napier’s (2015) argue that individuals recognize, notice, evaluate, and incorporate new values into their core mindset is a prerequisite to focus on new and emerging opportunities. They proposed five facets for assessing the mindsponge dimension. A better understanding of internet entrepreneurial intention to use technology products may benefit from considering the mindsponge dimension, where internet entrepreneurial intention is stimulated through the environmental factor – social support, and buffering mechanism – technology product imagination disposition and, intrinsic and extrinsic motivation. This study provides mechanism of filter in the mindsponge process of accepting the value of internet entrepreneurial. The stronger the disposition is, the higher the intrinsic and extrinsic motivation is, the more a person accept the internet entrepreneur idea (intention), which demonstrate a positive filtering process.

Furthermore, when a new technology is accepted by a person or it triggered our imagination, it entered the mindset through the buffer zone. People’s trust in future technology products may be meaningful to their entrepreneurial intention to use technology products. People’s trust may be formulated and evaluated from technical perspectives. Investigating this issue could be useful for furthering research and practice.

However, rejection also occurs in the process, as in this study social support negatively influences the intention, which means someone around you who started a business or supports you to start a business does not mean that an individual will accept the entrepreneurial value.

## Conclusion

In view of the rapid development of technology products, this study examined the formation of internet entrepreneurial intention to use technology products. This study makes three important contributions to theory and practice. First, technology products are suitable for internet entrepreneurship. The focus was on technology products, thus helping to deeply understand the formation of internet entrepreneurial intention to use technology products. Second, a multidimensional scale to assess technology product imagination disposition was developed and demonstrated. It provides a more comprehensive measure of technology product imagination disposition. Third, a model was developed based on SCT and SDT that includes technology product imagination disposition, intrinsic motivation, extrinsic motivation, and social support simultaneously. The results illustrate the important mediating roles of challenge, enjoyment, and outward motivation.

## Data Availability Statement

The raw data supporting the conclusions of this article will be made available by the authors, without undue reservation.

## Author Contributions

T-CH contributed to the conceptualization, methodology, investigation, verification, and writing – original draft. Y-JW contributed to the investigation, data analysis, visualization, and writing – original draft. H-ML contributed to the conceptualization, visualization, data analysis, verification, and writing – review and editing. All authors contributed to the article and approved the submitted version.

## Conflict of Interest

The authors declare that the research was conducted in the absence of any commercial or financial relationships that could be construed as a potential conflict of interest.

## Publisher’s Note

All claims expressed in this article are solely those of the authors and do not necessarily represent those of their affiliated organizations, or those of the publisher, the editors and the reviewers. Any product that may be evaluated in this article, or claim that may be made by its manufacturer, is not guaranteed or endorsed by the publisher.
