# Supplementary material for: What Drives Internet Entrepreneurial Intention to Use Technology Products? An Investigation of Technology Product Imagination Disposition, Social Support, and Motivation
Source: Front Psychol. 2022 Mar 16;13:829256. doi: 10.3389/fpsyg.2022.829256 (PMC8965873; doi:10.3389/fpsyg.2022.829256)
Supplement: Supplementary file 1 [file Data_Sheet_1.pdf]

Supplementary Material

What Drives Internet Entrepreneurial Intention to Use Technology Products? An Investigation of Technology Product Imagination Disposition, Social Support, and Motivation

Appendix A. Results for exploratory factor analysis of the Technology Product Imagination Disposition Scale.

**Item no.**	**Original item no.**	**Item**	**Factor**			
			Practical evaluation	Positive preference	Beyond reality	Positive value
3	4	I take the practicality of my imagined technology product into consideration.	.792			
5	6	I think of the presentation when imagining my technology product.	.780			
7	8	I take public acceptance of my technology product into account.	.639			
8	9	I imagine the appearance and functions of my technology product, the webpage layout, for instance.	.622			
10	12	I imagine my technology product by combining different features, such as material, software, hardware, and so on.	.592			
11	13	I evaluate the popularity of my imagined technology product.	.569			
2	3	I improve my imagination in many ways, such as reading fiction novels, attending creativity courses, and so on.		.797		
4	5	I find it pleasant to imagine technology products.		.714		
9	10	I consider that imagining a technology product can spice up our lives.		.644		
17	19	I find it interesting to imagine a technology product.		.588		

1	2	I can easily imagine technology products that do not exist.	.824	
6	7	I often think about changes in technology products today.	.738	
14	16	I often imagine different kinds of tools that humans will use in the future.	.582	
15	17	I expect customer demand for future technology products.	.567	
12	14	I consider that imagination can help to design a new technology product.		.804
13	15	I am able to connect things from my imagination as well as a virtual system and objects to reality.		.693
16	18	I consider that imagination can help to create a technology product.		.518

Note: Three items were deleted because the factor loadings were lower than 0.5. These items were the following: 1. I can imagine the idea of combining more than two things (such as theme, software, or hardware technology) into new technology products. 2. When I encounter inconvenience when using technology products, I imagine many transformation methods (e.g., how apps are used). 3. When I imagine a technology product, I imagine how it is used.

Appendix B. Results for item analysis of the Technology Product Imagination Disposition Scale.

Factor	Original item no.	CR value	Item–total correlation	Corrected item–total correlation	Cronbach's alpha if item deleted
Practical evaluation	4	7.601***	0.659***	0.604	0.896
	6	7.384***	0.685***	0.647	0.895
	8	8.119***	0.641***	0.583	0.597
	9	7.664***	0.614***	0.546	0.898
	12	10.179***	0.719***	0.664	0.895
	13	8.513***	0.629***	0.583	0.897
Positive preference	3	8.903***	0.617***	0.543	0.899
	5	8.553***	0.687***	0.636	0.895
	10	8.418***	0.639***	0.589	0.897
	19	6.935***	0.666***	0.598	0.897
Beyond reality	2	5.925***	0.582***	0.514	0.900
	7	8.977***	0.655***	0.613	0.896
	16	7.277***	0.553***	0.500	0.900
	17	7.494***	0.630***	0.564	0.898
Positive value	14	6.597***	0.549***	0.481	0.900
	15	6.968***	0.660***	0.598	0.897
	18	3.788***	0.440***	0.374	0.903

Appendix C. Variables and items.

Variable	Item	Factor loading	Source
Technology product imagination disposition (CA = 0.929, CR = 0.938, AVE = 0.472)			
TPID1	I can easily imagine technology products that do not exist.	0.540	Lin (2019)
TPID2	I improve my imagination in many ways, such as reading fiction novels, attending creativity courses, and so on.	0.561	
TPID3	I take the practicality of my imagined technology product into consideration.	0.727	
TPID4	I find it pleasant to imagine technology products.	0.750	
TPID5	I think of the presentation when imagining my technology product.	0.786	
TPID6	I often think about changes in technology products today.	0.698	
TPID7	I take public acceptance of my technology product into account.	0.668	
TPID8	I imagine the appearance and functions of my technology product, the webpage layout, for instance.	0.624	
TPID9	I consider that imagining a technology product can spice up our lives.	0.697	
TPID10	I imagine my technology product by combining different features, such as material, software, hardware, and so on.	0.761	
TPID11	I evaluate the popularity of my imagined technology product.	0.706	
TPID12	I consider that imagination can help to design a new technology product.	0.708	
TPID13	I am able to connect things from my imagination as well as a virtual system and objects to reality.	0.701	
TPID14	I often imagine different kinds of tools that humans will use in the future.	0.643	

TPID15	I expect customer demand for future technology products.	0.677	
TPID16	I consider that imagination can help to create a technology product.	0.630	
TPID17	I find it interesting to imagine a technology product.	0.753	
**Social support** (CA = 0.937, CR = 0.945, AVE = 0.489)			
IS1	Supporters provide experience and tell me how to deal with problems in stressful situations.	0.671	Krause and Markides (1990)
IS2	Supporters suggest what I should do to solve the problem I am facing.	0.670	
IS3	When I have a problem, a supporter provides me with information that helps to clarify the problem.	0.699	
IS4	Supporters help me understand why I'm not doing well.	0.680	
IS5	When I have a problem, a supporter tells me whom to ask for help.	0.605	
IS6	Supporters analyze the way I deal with the problem without commenting on its quality.	0.598	
TS1	Supporters are willing to give me or lend me more than NT$1,000.	0.491	
TS2	Supporters give me or lend me what I need (materials and goods other than money).	0.650	
TS3	Supporters are willing to help me do what I need to do, such as making a film.	0.657	
ES1	Supporters help me when I am under stress.	0.771	
ES2	Supporters tell me that I can.	0.779	
ES3	Supporters show empathy to comfort me.	0.790	
ES4	Supporters listen to my inner feelings.	0.795	
ES5	Supporters show empathy when we have a heart-to-heart talk.	0.785	
ES6	Supporters try to cheer me up in a humorous and witty way.	0.708	
ES7	Supporters express their attention and concern for my life.	0.755	
ES8	When I have a problem, a supporter will go with me to find someone who can help me.	0.693	

ES9	Supporters keep secret what I talk about to them.	0.715	
**Challenge motivation** (CA = 0.801, CR = 0.864, AVE = 0.565)			
CH1	I enjoy tackling problems that are completely new to me.	0.752	Adapted from Amabile et al. (1994)
CH2	I like to try to solve the complex problems that internet entrepreneurship may encounter.	0.856	
CH3	The more difficult the entrepreneurial problem, the more I enjoy trying to solve it.	0.840	
CH4	Internet entrepreneurship can increase knowledge and skills.	0.558	
CH5	Curiosity is the main motivation that drives me to start an internet business.	0.715	
**Enjoyment motivation** (CA = 0.813, CR = 0.870, AVE = 0.575)			
E1	I want to know how well I can do in internet entrepreneurship.	0.791	Adapted from Amabile et al. (1994)
E2	I like to solve problems by myself.	0.622	
E3	The most important thing for me is to enjoy my entrepreneurial process.	0.838	
E4	As long as I feel that I have gained a new experience, I will be satisfied regardless of the results of internet entrepreneurship.	0.739	
E5	I am more comfortable when I can set myself goals for internet entrepreneurship.	0.785	
**Compensation motivation** (CA = 0.811, CR = 0.868, AVE = 0.570)			
C1	The income from internet entrepreneurship can motivate me.	0.742	Adapted from Amabile et al. (1994)
C2	If I participate in an internet business competition, I will realize my goal.	0.773	
C3	I will consider subsidies or bonuses provided by the government or a competition for internet entrepreneurship.	0.825	
C4	I realize that the government provides subsidies for internet entrepreneurship.	0.746	
C5	I care about the rewards for internet entrepreneurship.	0.682	

**Outward motivation** (CA = 0.797, CR = 0.845, AVE = 0.443)			
O1	I believe internet entrepreneurship can be recognized by others.	0.774	Adapted from Amabile et al. (1994)
O2	I hope other people understand how well I can do in internet entrepreneurship.	0.786	
O3	To me, success means doing better than other people.	0.618	
O4	I think it is meaningless if no one knows how well I've done.	0.551	
O5	I'm concerned about how other people are going to react to my ideas.	0.626	
O6	I care more about the sense of accomplishment I can get in this job than about what I do.	0.700	
O7	I'm worried about what others think of my choice of internet entrepreneurship.	0.561	
**Internet entrepreneurial intention to use technology products** (CA = 0.934, CR = 0.950, AVE = 0.792)			
IEI1	My career goal is to become an internet entrepreneur of technology products.	0.875	Liñán and Chen (2009)
IEI2	I will try my best to create and run my internet business based on technology products.	0.875	
IEI3	I decided to start my own internet business based on technology products in the future.	0.930	
IEI4	I have seriously considered starting an internet business based on technology products.	0.907	
IEI5	In the future, I am willing to start an internet business based on technology products.	0.860	

Note: CA: Cronbach's alpha; CR: composite reliability; AVE: average variance extracted.

References

Amabile, T.M., Hill, K.G., Hennessey, B.A., and Tighe, E.M. (1994). The Work Preference Inventory: assessing intrinsic and extrinsic motivational orientations. *Journal of Personality and Social Psychology* 66**,** 950-967.

Krause, N., and Markides, K. (1990). Measuring social support among older adults. *International Journal of Aging and Human Development* 30**,** 37-53.

Liñán, F., and Chen, Y.W. (2009). Development and cross-cultural application of specific instrument to measure entrepreneurial intentions. *Entrepreneurship Theory and Practice* 33**,** 593-617.

Lin, M.-H. (2019). *Developing the scale of technology imagination disposition and Its correlation with grit and with Internet addiction.* master's thesis, National Taiwan Normal University.
